# Supplementary material for: Community participation in the collaborative governance of primary health care facilities, Uasin Gishu County, Kenya
Source: PLoS One. 2021 Mar 31;16(3):e0248914. doi: 10.1371/journal.pone.0248914 (PMC8011762; doi:10.1371/journal.pone.0248914)
Supplement: S1 Checklist — (DOCX) [file pone.0248914.s001.docx]

**S1 Checklist.**

5*Personal guidelines for documents review on community engagement*

1. Look for all documents from national, county, sub county level and facilities
2. Understanding the formal policies or guidelines that support community participation at facility level, particularly those related to governance.
3. For the documents related to community participation, what do they say about community participation? Is what they say about community participation related to governance of PHCs?
4. For the documents related to governance of PHC facilities, do they mention community participation in anyway?
5. Review of minutes of health facility committee meeting and county and sub- county health management committee and evaluate for attendance, issues related to community, issues related to community participation in governance at primary health care facility level.

6*Personal guidelines for observation during meetings*

**Observation guideline for note taking of meetings and fora**

1. Who is the chair of the meeting?
2. Who are involved? Are all members present?
3. Working through the agenda: who speaks, who is silent…
4. How are the marginalized groups treated? Present their ideas
5. Formal and informal parts of the meeting, duration, seating, soliciting of ideas from different people, agenda setting – consensus?
