# Supplementary material for: Community participation in the collaborative governance of primary health care facilities, Uasin Gishu County, Kenya
Source: PLoS One. 2021 Mar 31;16(3):e0248914. doi: 10.1371/journal.pone.0248914 (PMC8011762; doi:10.1371/journal.pone.0248914)
Supplement: S1 File — (DOCX) [file pone.0248914.s002.docx]

**S1 File.**

3.*Respondents at collaborative Level*

**Question**

Individuals or groups volunteer, get selected or are appointed to represent the community in governance of services including at healthcare facility. It is anticipated that the representation will facilitate service delivery. I would like to know the how they operate like process of selection, the roles and responsibilities and the impact on performance of service delivery in the healthcare facility if any. I would also like to know whether there are mechanisms that take care of representation of marginalized groups. Give specific examples where possible.

**Questions**

1. How were you selected to represent the community?
2. What are your roles and responsibilities in governance of this healthcare facility?
3. Who are the other stakeholders or partners that also participate in governance of primary health care facilities?
4. In what ways do community members participate in governance of the health facilities? Can you remember any examples?
5. Can you tell me more about any legal documents that support community participation in governance of facilities?
6. What can you attribute as the contribution of the community to the performance of the facility, give specific examples where possible?
7. Tell me more about how you get the community issues and present them to the committee
8. Are there any challenges that you experience when carrying out your duties?
9. Is there anything else you want to tell me?
10. Thank you
