# Supplementary material for: Community participation in the collaborative governance of primary health care facilities, Uasin Gishu County, Kenya
Source: PLoS One. 2021 Mar 31;16(3):e0248914. doi: 10.1371/journal.pone.0248914 (PMC8011762; doi:10.1371/journal.pone.0248914)
Supplement: Attachment — Submitted filename: Response to Reviewers 06202020.docx [file pone.0248914.s003.docx]

| PONE-D-20-06799 Community participation in the collaborative governance of primary health care facilities in Uasin Gishu County Kenya PLOS ONE | |
| --- | --- |
| **A: Responses to the Academic Editor and concerns addressed** | |
| 1. Introduction in abstract section is too long. | Introduction in the abstract edited to reduce  length; abstract is now 316 words including subtitles |
| 2. It is need to more clarify multiple case study methodology in method section. | Clarified in methods section (page 8). We have included details of study design, study population, methods and management and analysis of data, pp. 7-10 |
| 3. How you ensured your study rigor and trustworthiness? | We have included a paragraph on reflexivity and research rigor, page 14-15 |
| 4. Study objective was not fully transparent and specific. So this issue leads to misclassification of results. Please clarify study objectives and then based on this clarification classify results section in case of facilitators, barriers, …. | We examined the implementation of collaborative governance with an interest in the selection process, roles and responsibilities of members, and challenges faced by participants. This is because good governance requires that process should be transparent and participative to allow for accountability, with the goal of effective performance. |
| 5. How you used both deductive and inductive analysis for emerging themes, and how you classified themes? This section needs more clarification. | Siddique’s framework on governance plus other literature provided the lens from which community participation in governance was examined. This posits that the attributes of governance include: accountability, transparency, participation, consensus orientation, the provision of information, and ensuring inclusion, regulation and oversight. Emerging themes were also coded and presented through thick description |
| 6. You started result section with election process, you need to classify result based on your study objectives. So this section may be referring to current process of collaborative governance and its process. | The study objectives and results are now consistent. The focus of this article is on the implementation of collaborative governance at primary health care facilities, looking at the structures in place for operations such as the selection processes for membership of the committee, the demarcation and undertaking of roles and responsibilities, and the challenges faced in Uasin Gishu County. In addition, we address the exercise of power, at the time of the election and in proceedings of meetings. |
| 7. Please provide thematic table or theme tree, so this enables reader to find themes and sub-themes easily and the relation between themes. | A thematic table has been provided as supporting information as Table 3 page 14-15 |
| 8. Please provide the institutional email address of the Corresponding Author. | This has been provided in the manuscript |
| 9. Please include additional information regarding the observation and interview guidelines used in the study and ensure that you have provided sufficient details that others could replicate the analyses. For instance, if you developed observation and interview guidelines as part of this study and it is not under a copyright more restrictive than CC-BY, please include a copy, in both the original language and English, as Supporting Information. | Interview and observation guides were developed by the first author in collaboration with the other authors who are the supervisors. The questions were informed by literature review on governance. These instruments are not under any restrictions and are provided as supporting information for readers |
| 10. Please provide additional details regarding participant consent. In the ethics statement in the Methods and online submission information, please ensure that you have specified whether consent was informed. | Ethics (Page 15) and online submission ethics statements are provided |
| 11. Please include your tables as part of your main manuscript and remove the individual files. Please note that supplementary tables (should remain/ be uploaded) as separate "supporting information" files | Tables have been included as part of the main manuscript |
| 12. Please amend either the title on the online submission form (via Edit Submission) or the title in the manuscript so that they are identical. | The title on the online submission has been amended |
| 13. Thank you for stating the following in your Competing Interests section: "none declared” Please complete your Competing Interests on the online submission form to state any Competing Interests. If you have no competing interests, please state "The authors have declared that no competing interests exist.", as detailed online in our guide for authors at <http://journals.plos.org/plosone/s/submit-now>. This information should be included in your cover letter; we will change the online submission form on your behalf. Please know it is PLOS ONE policy for corresponding authors to declare, on behalf of all authors, all potential competing interests for the purposes of transparency. PLOS defines a competing interest as anything that interferes with, or could reasonably be perceived as interfering with, the full and objective presentation, peer review, editorial decision-making, or publication of research or non-research articles submitted to one of the journals. Competing interests can be financial or non-financial, professional, or personal. Competing interests can arise in relationship to an organization or another person. Please follow this link to our website for more details on competing interests: <http://journals.plos.org/plosone/s/competing-interests> | The authors declare that no competing interests exist |
| 14. We note that you have indicated that data from this study are available upon request. PLOS only allows data to be available upon request if there are legal or ethical restrictions on sharing data publicly. For information on unacceptable data access restrictions, please see <http://journals.plos.org/plosone/s/data-availability#loc-unacceptable-data-access-restrictions>. | Data can be available upon request, as explained in the cover letter. The restrictions relate to anonymity of the facilities and the requirement that any new investigator wishing to access data must apply for ethics clearance. I have elaborated this in the cover letter. |
|  |  |
| **B. In your revised cover letter, please address the following prompts:** | We have addressed these issues in the cover letter but summaries our responses also below |
|  |  |
| 15. If there are ethical or legal restrictions on sharing a de-identified data set, please explain them in detail (e.g., data contain potentially identifying or sensitive patient information) and who has imposed them (e.g., an ethics committee). Please also provide contact information for a data access committee, ethics committee, or other institutional body to which data requests may be sent. | Data can be provided to the editor for the purposes of transparency of the study if need be. However, as explained in the cover letter, governance is a sensitive topic and qualitative data is difficult to de-identify the data completely. Data from this study can be availed through approving ethics committee -Institutional ethics and research Committee (IREC) Moi teaching and referral Hospital (MTRH) and Moi University, College of health Sciences. To access the data one can, write to the board chair, IREC, P.O. Box 3-30100, Eldoret, Kenya. Email: [irecmtrh@gmail.com](mailto:irecmtrh@gmail.com) or [contact@irec.or.ke](mailto:contact@irec.or.ke). Contact person is Ms Cathrine Okwiri. |
|  |  |
| 16. If there are no restrictions, please upload the minimal anonymized data set necessary to replicate your study findings as either Supporting Information files or to a stable, public repository and provide us with the relevant URLs, DOIs, or accession numbers. Please see <http://www.bmj.com/content/340/bmj.c181.long> for guidelines on how to de-identify and prepare clinical data for publication. For a list of acceptable repositories, please see <http://journals.plos.org/plosone/s/data-availability#loc-recommended-repositories>. | For reasons set out above, data cannot be uploaded and made publicly available. |
|  |  |
| We will update your Data Availability statement on your behalf to reflect the information you provide. | Thank you |
|  |  |
| 17. Your ethics statement must appear in the Methods section of your manuscript. If your ethics statement is written in any section besides the Methods, please move it to the Methods section and delete it from any other section. Please also ensure that your ethics statement is included in your manuscript, as the ethics section of your online submission will not be published alongside your manuscript. | Ethics statement has been revised and included in the manuscript (Page 15) |
| **Reviewer's Responses to Questions**  **C. Comments to the Author** | |
| 18. Is the manuscript technically sound, and do the data support the conclusions?  The manuscript must describe a technically sound piece of scientific research with data that supports the conclusions. Experiments must have been conducted rigorously, with appropriate controls, replication, and sample sizes. The conclusions must be drawn appropriately based on the data presented.  Reviewer #1: Partly  Reviewer #2: Yes |  |
| 19. Has the statistical analysis been performed appropriately and rigorously?  Reviewer #1: Yes  Reviewer #2: I Don't Know |  |
| 20. Have the authors made all data underlying the findings in their manuscript fully available?  The [PLOS Data policy](http://www.plosone.org/static/policies.action#sharing) requires authors to make all data underlying the findings described in their manuscript fully available without restriction, with rare exception (please refer to the Data Availability Statement in the manuscript PDF file). The data should be provided as part of the manuscript or its supporting information, or deposited to a public repository. For example, in addition to summary statistics, the data points behind means, medians and variance measures should be available. If there are restrictions on publicly sharing data—e.g. participant privacy or use of data from a third party—those must be specified.  Reviewer #1: No  Reviewer #2: No |  |
| 21. Is the manuscript presented in an intelligible fashion and written in standard English?  22. PLOS ONE does not copyedit accepted manuscripts, so the language in submitted articles must be clear, correct, and unambiguous. Any typographical or grammatical errors should be corrected at revision, so please note any specific errors here.  Reviewer #1: Yes  Reviewer #2: Yes |  |
| **Reviewer Comments to the Author** | |
| 23. Please use the space provided to explain your answers to the questions above. You may also include additional comments for the author, including concerns about dual publication, research ethics, or publication ethics. (Please upload your review as an attachment if it exceeds 20,000 characters)  24. Reviewer #1: The author utilized a multiple case study methodology, selected five primary health care facilities purposively. Examined the implementation of collaborative governance in primary health care facilities in Uasin Gishu County, Western Kenya, under the policy of devolved governance of 2013. However, what is the significance of the research? How to choose the interviews to ensure representative？ Some problems in the citation of references, such as 16-20 17 21，and also a little outmoded.   25. Reviewer #2: Authors worked on an interesting topic. However, In my opinion this paper needs an extensive modifications by the authors. General comment: Make a consistent writing style in the whole article. In introduction section you started all paragraphs, leaving some space as contrary in other sections. Focus on editing and some minor grammatical mistakes in the reviewed version. Introduction: The first line (introductory) seems to have no link with the next sentences in first paragraph. Modify the first few lines to make a clear introduction of the topic. In addition, I felt lack of details about the significance of community role and participation in primary healthcare. Therefore, the authors may add few lines. Don't mention the place of tables like this: TABLE ONE ABOUT HERE..........it creates confusion. We already know that you placed the tables at the end. In the third paragraph the authors wrote........In 2010, Kenya initiated.....before this line, you can add few lines about the primary healthcare system in Kenya as this is the topic you focused in your study. Make a consistent style throughout the paper. In introduction section in the last lines..........."we examine in-depth the implementation of ......." use the past tense as you did in the methods section. Also review this sentence to make it more comprehensible and structured. Methods: In study setting, you wrote "This county was selected purposively for its strategic location in the Western region of Kenya, in providing health services"...please mention why western region is important in this regard? In interview section briefly describe the topic of the question you asked during interviews. Clearly mention the variables of your study in the methods section. Conclusion: Add some recommending remarks for other countries based on your study. | Significance of the study has been provided on Page 7, and this is reiterated in the background and in the discussion. We believe that the article will help inform strategies for the more effective implementation of community governance and this should ultimately strengthen social accountability, health outcomes and development  Interviewees were selected to ensure representation, as explained on Page 12-13 of the manuscript. Citation has been revised accordingly.  The paper has been revised extensively including the introduction.  Clauses on the tables have been removed  A paragraph has been inserted introducing primary health care in Kenya (Page 4)  The reason for the selection of Uasin Gishu County for the study has been provided (Page 10)  Variables under study are explained on page 13 |
| 26. PLOS authors have the option to publish the peer review history of their article. If published, this will include your full peer review and any attached files.   If you choose “no”, your identity will remain anonymous but your review may still be made public.   **Do you want your identity to be public for this peer review?** For information about this choice, including consent withdrawal, please see our [Privacy Policy](https://www.plos.org/privacy-policy).  Reviewer #1: No  Reviewer #2: No  [NOTE: If reviewer comments were submitted as an attachment file, they will be attached to this email and accessible via the submission site. Please log into your account, locate the manuscript record, and check for the action link "View Attachments". If this link does not appear, there are no attachment files to be viewed.]   While revising your submission, please upload your figure files to the Preflight Analysis and Conversion Engine (PACE) digital diagnostic tool, <https://pacev2.apexcovantage.com/>. PACE helps ensure that figures meet PLOS requirements. To use PACE, you must first register as a user. Registration is free. Then, login and navigate to the UPLOAD tab, where you will find detailed instructions on how to use the tool. If you encounter any issues or have any questions when using PACE, please email us at [figures@plos.org](mailto:figures@plos.org). Please note that Supporting Information files do not need this step. | This is relevant to the reviewers and not to the authors of this manuscript. |
