# Supplementary material for: Community participation in the collaborative governance of primary health care facilities, Uasin Gishu County, Kenya
Source: PLoS One. 2021 Mar 31;16(3):e0248914. doi: 10.1371/journal.pone.0248914 (PMC8011762; doi:10.1371/journal.pone.0248914)
Supplement: Attachment — Submitted filename: RESPONSE TO REVIEWERS.docx [file pone.0248914.s004.docx]

| ITEM BY ITEM REVISIONS | |
| --- | --- |
| PONE-D-20-06799R1 Community participation in the collaborative governance of primary health care facilities, Uasin Gishu County. Kenya PLOS ONE | |
| 1. Please correct and complete the title of table 3 as informative format. Thematic table is part of your result not method section, and must be presented in result section. Also, it appears correct table as right to left format (code, category and theme). | Table has been reviewed and moved to results section page 25-27 |
| 2. The coding process in its current format is not satisfactory. Codes in some case raised as quotes and in some cases as codes. | Edited to improve as presented in page 25-27 |
| a. categories and themes were not related logically and conceptually. You can find a useful structure to coding process in following references: | These articles have been reviewed and knowledge has been used in improving the thematic table. They have also been referenced |
| - de Casterlé BD, GasAtmans C, Bryon E, Denier Y. QUAGOL: A guide for qualitative data analysis. International journal of nursing studies. 2012 Mar 1;49(3):360-71. |  |
| - Graneheim UH, Lundman B. Qualitative content analysis in nursing research: concepts, procedures and measures to achieve trustworthiness. Nurse education today. 2004 Feb 1;24(2):105-12. |  |
| - Iezadi, S., Tabrizi, J.S., Ghiasi, A. et al. Improvement of the quality payment program by improving data reporting process: an action research. BMC Health Serv Res 18, 692 (2018). <https://doi.org/10.1186/s12913-018-3472-4> |  |
| 3. In order to provide a more complete information to our readers on the topic, we would like to emphasize the importance to cross referencing very recent material on the same topic published in "PLoS ONE ". Therefore, it would be highly appreciated if you would check the contents published in the last two years of "PLoS ONE" (<https://journals.plos.org/plosone/>) and add all material relevant to your article to the reference list. | Checked relevant articles from PLOS website and referenced them in them intext and at reference section |
| While revising your submission, please upload your figure files to the Preflight Analysis and Conversion Engine (PACE) digital diagnostic tool, <https://pacev2.apexcovantage.com/>. PACE helps ensure that figures meet PLOS requirements. To use PACE, you must first register as a user. Registration is free. Then, login and navigate to the UPLOAD tab, where you will find detailed instructions on how to use the tool. If you encounter any issues or have any questions when using PACE, please email PLOS at [figures@plos.org](mailto:figures@plos.org). Please note that Supporting Information files do not need this step. | Earlier uploaded and checked |
