# Supplementary material for: Community participation in the collaborative governance of primary health care facilities, Uasin Gishu County, Kenya
Source: PLoS One. 2021 Mar 31;16(3):e0248914. doi: 10.1371/journal.pone.0248914 (PMC8011762; doi:10.1371/journal.pone.0248914)
Supplement: Attachment — Submitted filename: Response to reviewer.docx [file pone.0248914.s005.docx]

**Dear Editor**

***Thank you for the concerns raised.***

***The concerns bellow has been addressed in the manuscript in page 24- to 26***

In table 4 use bullets to shows each code. Also affiliate every code to each sub theme (replace with category) and every sub theme to each theme. Use same wording in table as text to represent codes, sub theme and themes.

| Code | sub theme | theme |
| --- | --- | --- |
| - Code 1 - Code 2 - Code 3 | sub theme | theme |
| - Code 1 - Code 2 - Code 3 | sub theme |  |

***Thank You***

***Regards***

***Jackline Sitienei***

***On Behalf of the authors***
